# Genomic and epidemiologic evidence for attenuated virulence in *Salmonella* Montevideo associated with cattle and beef products in the United States

**DOI:** 10.3389/fmicb.2026.1837237

**Published:** 2026-06-02

**Authors:** Ruixi Chen, Linghuan Yang, Martin Wiedmann, Renato H. Orsi

**Affiliations:** Department of Food Science, Cornell University, Ithaca, NY, United States

**Keywords:** BEAST analysis, beef, cattle, comparative genomics, evolution, molecular clock analysis, *Salmonella* Montevideo, virulence attenuation

## Abstract

*Salmonella enterica* serovar Montevideo (*S*. Montevideo) is frequently isolated from cattle, their products, and related environments, yet rarely causes human illness through these sources, suggesting variation in human virulence and niche adaptation among its lineages. Building on our previous identification of a predominant *S*. Montevideo phylogenetic group comprising 12 clades with distinct ecological associations, we conducted comparative genomic and evolutionary analyses to characterize clades differing in human association and source adaptation. We identified one non-human-associated (NHA) clade (clade 10, cattle-adapted) and three human-associated (HA) clades (clade 7, non-animal-environment-adapted; clades 3 and 6, lacking source specificity), all four of which exhibited open pan-genomes, indicating ongoing genetic exchange. Of the genes significantly overrepresented in the HA clades relative to the NHA clade, 35%−66% were carried on mobile genetic elements. In contrast, all five virulence factors enriched in HA clades were chromosomally encoded. Compared to HA clades, the NHA clade showed a 7–19-fold enrichment in premature stop codons in genes encoding virulence factors, including genes involved in epithelial invasion and systemic infection, suggesting functional loss linked to host adaptation and reduced virulence. Finally, evolutionary reconstruction revealed that clade 10 emerged most recently and evolved the fastest, reflecting rapid host adaptation, whereas clade 7 is ancient and demonstrated genomic stability indicative of long-term environmental persistence. Clades 3 and 6 displayed intermediate evolutionary rates, with clade 3 encompassing two recently emerged sub-clades exhibiting signatures of clonal expansion. Hence, our study underscores the need for a risk-based approach to control for *Salmonella* in agricultural animals and their derived food products to generate a more efficient and significant public health impact.

## Introduction

*Salmonella enterica* subspecies *enterica* serotype (*S*.) Montevideo is often isolated from cattle, beef, and cattle-related environments (Blau et al., 2005; USDA-APHIS, 2005; Bosilevac et al., 2009; Valenzuela et al., 2017; Craig et al., 2024). In 2002, *S*. Montevideo was the second most commonly isolated serotype from dairy operation cattle feces (11.9% of positive samples), and the most commonly isolated serotype from dairy cattle herds in the United States (US, 8.3%) (USDA-APHIS, 2005). Between July 2005 and June 2007, *S*. Montevideo was the most commonly isolated serotype (21% of the positive samples) from commercial ground beef in a study in the US (Bosilevac et al., 2009). *S*. Montevideo was the fourth most prevalent serotype isolated from bovine samples submitted to veterinary diagnostic laboratories in central New York between 2007 and 2021 (6% of *Salmonella*-positive bovine fecal and gastrointestinal samples; Craig et al., 2024) and in Wisconsin between 2006 and 2015 (8% of *Salmonella*-positive bovine fecal and tissue samples; Valenzuela et al., 2017).

Nevertheless, outbreaks linked to beef (Jackson et al., 2013; Laufer et al., 2015; Canning et al., 2023; Marshall et al., 2024) and dairy products (Jackson et al., 2013; Gould et al., 2014) are rarely associated with *S*. Montevideo. Exceptions to these include a 1995 *S*. Montevideo outbreak in New Mexico linked to consumption of beef jerky (Centers for Disease Control and Prevention, 1995), a 2006 outbreak linked to consumption of cheese made from raw milk in France in 2006 (Dominguez et al., 2009), and two outbreaks linked to consumption of cheese made from pasteurized milk in the US (Gould et al., 2014), which may suggest a non-dairy source in these two outbreaks as pasteurization would have killed all *Salmonella* in the milk used in the cheese making. Conversely, *S*. Montevideo has been linked to several outbreaks linked to other sources. For example, between 2010 and 2025, *S*. Montevideo strains have been implicated in outbreaks linked to pistachio (Haendiges et al., 2021), red and black pepper (Gieraltowski et al., 2013), raw sprouts (Centers for Disease Control and Prevention, 2018), and live backyard poultry (Gaffga Nicholas et al., 2012; Centers for Disease Control and Prevention, 2012). The low number of *S*. Montevideo outbreaks linked to beef and dairy products suggests that despite their high prevalence in beef, cattle, and cattle-related environments, at least some *S*. Montevideo strains found in these environments may be impaired in their ability to cause human illness.

In a previous study, we showed that *S*. Montevideo is a polyphyletic serotype, and its main lineage (*S*. Montevideo A) comprises 12 clades with distinct ecological associations, genomic diversity, and likelihood of causing human disease (Chen et al., 2024). For instance, one clade (*S*. Montevideo A clade 10), which comprised the largest number of isolates in the NCBI Pathogen Detection (PD) database, was predominantly isolated from cattle-related sources and contained relatively few human isolates. Conversely, another clade (*S*. Montevideo A clade 7) was predominantly isolated from human sources and was significantly associated with non-animal natural environments, such as irrigation water and sediment.

In this study, we applied a formal statistical approach to delineate clades within *S*. Montevideo A that exhibit a significant over- or underrepresentation of human isolates, followed by comparative genomic and evolutionary analyses to elucidate the interplay among the presumptive hypo- or hypervirulence, niche adaptation, and the evolution of the target clades. The key findings of this work emphasize the need for interventions that use a risk-based approach and take into consideration the *Salmonella* subtype, instead of considering all *Salmonella* as having the same likelihood to cause human illness, to improve the impact of these interventions to public health.

## Materials and methods

### Identification of human-associated and non-human-associated clades within the predominant phylogenetic lineage of *S*. Montevideo

**Figure 1:**
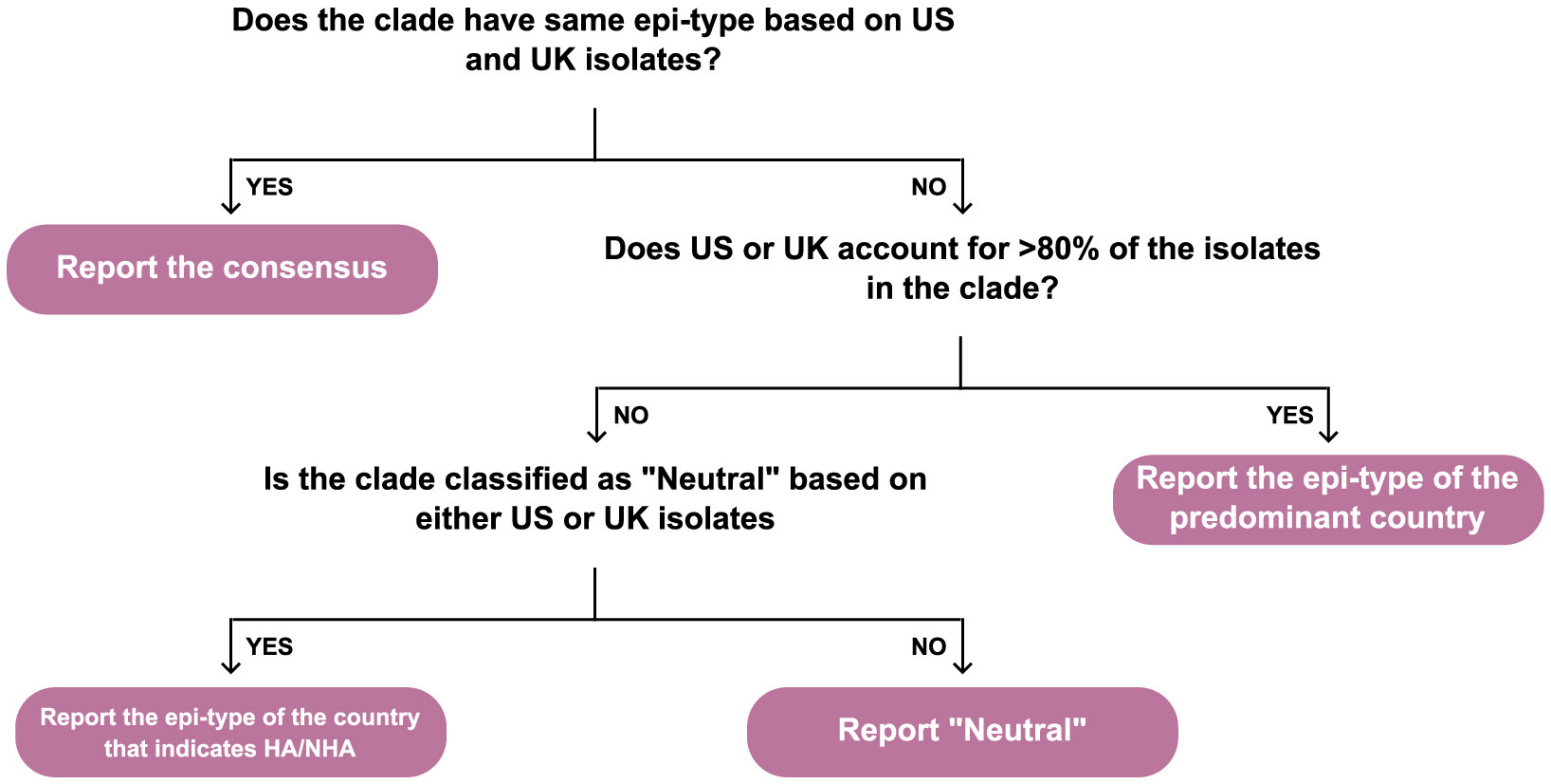
Decision framework for assigning final epidemiological types (epi-types) to *S*. Montevideo A clades. For each clade, epi-types were first determined independently using isolates from the US and the UK. If both countries indicated the same classification, the consensus was assigned. If classifications differed, the epi-type of the predominant country (>80% of isolates within the clade) was assigned. If neither country predominated, clades were classified as neutral unless one country indicated a non-neutral classification [i.e., human-associated (HA) or non-human-associated (NHA)], in which case that classification was assigned.

Whole-genome sequencing (WGS) data and associated metadata, such as collection time, geographical location, 7-gene multi-locus sequence type (MLST), and antimicrobial resistance (AMR) profiles, for all *S*. Montevideo A isolates were obtained from datasets reported by Chen et al. (2024). To assess the association of *S*. Montevideo A clades with human salmonellosis cases, the odds ratio (OR) for human isolates (i.e., the odds of human isolates within a given clade relative to the odds of human isolates among all *Salmonella* isolates outside the clade) was calculated for each clade, followed by a one-sided Fisher's exact test to determine statistical significance, with *p*-values adjusted for multiple comparisons using the Benjamini-Hochberg (BH) method. Using only isolates from the US and the United Kingdom (UK), all clades were subsequently classified into three epidemiology types (“epi-types”) based on their association with human salmonellosis cases: (i) human-associated (“HA”; OR > 2 and *p*-value <0.05), (ii) non-human-associated (“NHA”; OR <0.5 and *p*-value <0.05), and (iii) neutral (0.5 ≤ OR ≤ 2 and/or *p*-value ≥ 0.05). The selected OR thresholds correspond to at least a two-fold change in odds and, when combined with statistical significance, are commonly used to define meaningful associations in epidemiological analyses (Stafford et al., 2008; Domingues et al., 2012; Ziehm et al., 2013). Notably, the US and the UK together accounted for 81% of all *Salmonella* isolates in the NCBI PD database (accessed August 31, 2021), with the proportion of human isolates varying considerably between the two countries (70% in the US and 93% in the UK). To mitigate ascertainment biases arising from geographical associations, for each clade, epi-types were independently determined based on US and UK isolates, respectively, and the final epi-type was assigned following a pre-defined decision framework (Figure 1): if both countries indicated the same classification, it was retained; if classifications differed, the epi-type of the predominant country (>80% of isolates) was assigned; if neither country predominated, clades were classified as neutral unless only one country indicated a non-neutral classification, in which case that classification was used. The resulting HA and NHA clades represented the primary focus of all downstream analyses. Importantly, although the epi-type assignment relied solely on the US and UK isolates, all available global isolates were included in the downstream analyses.

### Selection of isolates from HA and NHA clades

To determine the number of isolates to include from each HA and NHA clade for the comparative genomic analysis, a sample size calculation was performed using OpenEpi (Dean et al., 2013) with the following parameters: power = 80%, two-sided confidence level = 95%, proportion of unexposed with outcome (e.g., HA isolates lacking a potential virulence factor) = 30%, and OR (e.g., the odds of HA isolates among all isolates carrying a potential virulence factor divided by the odds of HA isolates among those without it) = 4. This analysis indicated that 40 isolates were required from each clade for the comparative genomic analysis. Forty isolates were then selected from each clade using the following procedure: (i) single nucleotide polymorphism (SNP) clusters within each clade were ranked based on the total number of isolates, from highest to lowest, considering singletons as SNP clusters with only one isolate; (ii) from the top 40 SNP clusters, the isolate with the fewest contigs was selected from each SNP cluster; (iii) if the total number of SNP clusters within a clade was <40, the isolate with the second fewest contigs was selected from each SNP cluster, starting from the top of the ranked list, continuing down until the dataset was complete. Importantly, this sampling strategy was designed to capture genomic diversity at the clade level, rather than to reflect the frequency of dominant clonal lineages, thereby increasing the likelihood of identifying clade-wide genomic features.

### Reference-free core SNP variant calling and phylogenetic analyses

To assess the phylogenetic relationship across the HA and NHA clades, kSNP4 v 4.1 (Hall and Nisbet, 2023) was used to identify core SNPs among the representative isolates from all clades. A reference isolate from *S*. Oranienburg was included as the outgroup, as this serovar has been previously reported to be phylogenetically closely related to *S*. Montevideo A (Chen et al., 2024). This k-mer-based method is well-suited for capturing variation across genetically diverse isolates across clades without introducing biases associated with reference genome selection. The optimal k-mer size of 19 was estimated using Kchooser. The resulting core SNP alignment was subsequently used to reconstruct a maximum likelihood phylogeny using RAxML v 8.2.13 (Stamatakis, 2014), specifying the GTRCATX model with Lewis ascertainment bias correction and 1,000 bootstrap replicates. The phylogenetic tree was visualized and annotated using Interactive Tree of Life (iTOL) (Letunic and Bork, 2024).

### Pan-genome estimation and openness assessment

For each HA and NHA clade, 40 representative isolates were selected using stratified random sampling based on SNP cluster size to ensure proportional representation of dominant and rare genetic lineages. Genome assemblies of the selected isolates were annotated in general feature format (GFF3) using Prokka v 1.14.5 (Seemann, 2014), applying standard settings for Gram-negative microorganisms. Panaroo v 1.4.0 (Tonkin-Hill et al., 2020) was used to infer the pangenome among the representative isolates, taking the GFF3 files as input. To assess the openness of the pan-genome, a rarefaction curve was constructed with 1,000 permutations using the micropan v 2.1 package (Snipen and Liland, 2015) in R Statistical Programming Environment (R) v 4.3.2 (R Core Team, 2021). The resulting curve was fitted with the Heaps law model (Tettelin et al., 2008), defined by the following equation:


$$\begin{array}{l} \text{n}=\text{k}\times \;{\text{N}}^{\gamma } \end{array}$$

where *N* represents the number of genomes, *n* represents the expected number of genes in the pan-genome, and *k* and γ are free parameters. The values of *k* and γ were then extracted from the fitted model, and an alpha value was calculated using the following equation:


$$\begin{array}{l} \text{alpha}=\text{1}-\gamma \end{array}$$

where the pan-genome was considered closed if alpha > 1 and open if alpha <1 (Tettelin et al., 2008).

### Identification of accessory genes likely associated with enhanced or reduced human virulence

For each pair of NHA and HA clades, the pan-genome among representative isolates from both clades were estimated using Panaroo v 1.4.0 (Tonkin-Hill et al., 2020) with the same setting as described in the section “Pan-genome estimation and openness assessment.” To gain further insights into the function of genes comprising the pan-genome, especially those annotated by Prokka as encoding hypothetical proteins, InterProScan v 5.53-87.0 (Jones et al., 2014) was used to retrieve additional annotations for each putative gene family. To identify genes that likely contribute to an enhanced or reduced human virulence, a pangenome-wide association study (pan-GWAS) was performed using Scoary v 1.6.16 (Brynildsrud et al., 2016) to identify accessory genes overrepresented among the HA or NHA clades (“HA genes” and “NHA genes,” respectively); the statistical significance was assessed using Fisher's exact tests with BH correction for multiple testing. Genes with an adjusted *p*-value <0.05 were considered statistically significant. To identify genes that are likely to represent established virulence factors, BLAST+ v 2.13.0 (Camacho et al., 2009) was used to query the HA and NHA genes against a custom database, comprising reference sequences from VFDB (Liu et al., 2022), PATRIC_VF (Wattam et al., 2014), and Victors (Sayers et al., 2019), with cutoffs of >90% sequence identity and >90% query coverage; the databases were downloaded on September 20, 2022.

### Identification of premature stop codons likely associated with enhanced or reduced human virulence

For each pair of NHA and HA clades, a k-mer-based SNP variant calling was performed using kSNP4 v 4.1 (Hall and Nisbet, 2023) to identify core SNPs among the representative isolates. The complete genome of a representative isolate from the NHA clade (GenBank accession number: GCA_005576575.1) was designated as the reference for core SNP annotation. The -vcf flag was specified for generating a Variant Calling Format file to store genetic variant data. Core SNPs leading to premature stop codons (PMSCs; i.e., nonsense mutations likely leading to dysfunctional proteins) were subsequently identified. A similar GWAS analysis was then performed to identify PMSCs overrepresented among either the HA or NHA clade (“HA PMSCs” and “NHA PMSCs,” respectively), using the same approach described above. Genes harboring HA or NHA PMSCs were examined to identify those likely representing established virulence factors using the same approach described above.

### *In silico* classification of genes based on genomic origin

To assess the genomic origins of putative gene families within the pan-genome, individual genes from the genome assembly of each representative isolate were classified into four categories: (i) Chromosomal, (ii) Plasmid-borne, (iii) Prophage-borne, and (iv) Undetermined. Specifically, for each isolate, Platon v 1.6 (Schwengers et al., 2020) was used to identify genome assembly contigs that likely originate from plasmids, while PHASTER (Zhou et al., 2011; Arndt et al., 2016) was used to identify regions within contigs likely derived from prophages. Identified prophage regions belonging to all categories (i.e., “intact,” “questionable,” or “incomplete”) were included. Custom scripts were then used to parse the GenBank file generated by Prokka, extracting contig and coordinate information for each gene, which was subsequently cross-referenced with the Platon and PHASTER results to determine the genomic origin of individual genes; genes were considered to be in a plasmid or in a prophage if 100% of the gene length was enclosed within these two elements. A gene was classified as “Chromosomal” if it was not associated with plasmids or prophages, “Plasmid-borne” if associated with plasmids but not prophages, “Prophage-borne” if associated with prophages but not plasmids, and “Undetermined” if it could not be unambiguously assigned to the three preceding categories. The “refound” gene copies identified by Panaroo were also classified as “Undetermined.” Finally, the genomic origin of each putative gene family was represented by the distribution of its member genes across these origin categories.

### Evolutionary analysis

To elucidate the evolutionary trajectories of the HA and NHA clades, evolutionary analyses were performed to (i) estimate key evolutionary parameters [e.g., time to most recent common ancestor (TMRCA), evolutionary rate] and (ii) reconstruct time-scaled phylogenies and past population dynamics. For each clade, 100 representative isolates were selected using stratified random sampling based on the collection year. Specifically, isolates within each clade were grouped into four temporal bins (before 2007, 2007–2011, 2012–2016, and 2017–2021), from which 25 isolates were randomly selected per bin. Raw sequence reads of the selected isolates were retrieved from the Sequence Read Archive (SRA) (Leinonen et al., 2011) using SRA Toolkit v 3.1.1 (https://github.com/ncbi/sra-tools) and pre-processed with fastp v 0.12.4 (Chen et al., 2018) to remove low-quality reads and trim sequencing adaptors. A referenced-based SNP variant calling was performed using Snippy v 4.6.0 (https://github.com/tseemann/snippy), specifying the genome assembly containing the fewest contigs (GenBank accession numbers: GCA_008957465.1 for clade 3 and GCA_006689685.1 for clade 6) as the reference. Recombination sites within the resulting whole-genome alignment were identified and removed using Gubbins v 3.4 (Croucher et al., 2015). The recombination-free alignment was then processed with snp-sites v 2.5.1 (Page et al., 2016) to (i) quantify the number of invariant sites for each nucleotide type (i.e., A, C, G, and T) and (ii) extract the variant sites to produce a core SNP alignment among the representative isolates.

The dataset for each clade was subjected to the Date-Randomization Test (DRT) to assess whether it contained sufficient temporal signal to reliably estimate evolutionary rates, divergence times, as well as other evolutionary parameters using molecular clock models. Specifically, BEAUti v 2.5.2 (Bouckaert et al., 2019) was used to generate an XML configuration file based on the dataset, specifying the HKY substitution model, the strict molecular clock, and the coalescent constant population size prior. To correct for ascertainment bias resulting from the use of variant sites only, the counts of invariant sites for each nucleotide type were manually incorporated into the XML file (https://groups.google.com/g/beast-users/c/QfBHMOqImFE). The initial clock rate was set to 2.1 × 10^7^ substitutions/site/year. Subsequently, the TipDatingBeast v 1.1-0 package (Rieux and Khatchikian, 2017) in R was used to generate five date-randomized variants of the original dataset and their corresponding XML files, in which sampling dates were randomly assigned to isolates, thereby breaking the association between substitutions and time. BEAST v 2.5.2 (Bouckaert et al., 2019) was then used to estimate the substitution rates for both the original and date-randomized datasets. The results were visualized using TipDatingBeast, and the dataset was considered to have passed DRT if the substitution rate estimated from the original dataset fell outside the 95% credible intervals of those from the date-randomized datasets (Duchêne et al., 2015).

For each clade, BEAST v 2.5.2 was used to run a Markov Chain Monte-Carlo (MCMC) analysis for estimating the posterior probability distributions of genealogical and demographic parameters, applying the optimized settings from a previous study (Chen et al., 2024), including (i) a substitution model enforcing identical rates for AC/GT transversions and AG/CT transitions, (ii) Gamma rate heterogeneity, (iii) the lognormal relaxed molecular clock, and (iv) the coalescent Bayesian Skyline model. To ensure robustness and reproducibility, each analysis involved three independent MCMC chains with distinct random seeds, specifying a chain length of 200 million generations and sampling every 10,000 generations. LogCombiner v 2.5.2 (Bouckaert et al., 2019) was used to merge the log files from the three independent MCMC chains, discarding the first 10% of iterations from each chain as burn-in. Tracer v 1.7.2 (Rambaut et al., 2018) was used to assess the effective sample sizes (ESSs) of run statistics and to visually compare trace patterns across individual MCMC chains. Parameter estimates were considered reliable if (i) the ESS exceeded 100 for individual chain and 200 when all three chains were merged, and (ii) the trace plots from different chains overlapped without showing distinct trends. LogCombiner was used to merge the tree files from the three independent MCMC chains, discarding the first 10% of iterations from each as burn-in. The merged tree file was analyzed using TreeAnnotator v 2.5.2 (Heled and Bouckaert, 2013) to produce a maximum clade credibility tree (i.e., the time-scaled phylogeny). The resulting tree was visualized and annotated using the ggtree v 3.10.0 (Yu et al., 2018; Yu, 2020) and treeio v 1.26.0 (Wang et al., 2020; Yu, 2022) packages in R, incorporating metadata for the representative isolates used to construct the phylogeny, such as collection year, geographical location, and number of antimicrobial resistance (AMR) classes, obtained from Chen et al. (2024). Finally, Tracer was used to generate a Bayesian Skyline plot (BSP) based on the merged log and tree files to infer past population dynamics.

## Results

### The major phylogenetic lineage of *S*. Montevideo comprises clades with significant over- or underrepresentation of human isolates

Our previous study revealed that *S*. Montevideo is polyphyletic, comprising four distinct phylogenetic lineages, designated *S*. Montevideo A through D (Chen et al., 2024). Among these, *S*. Montevideo A represented the predominant lineage, encompassing 99.7% of the isolates and comprising 12 clades with considerably variable odds ratios of human isolate association, clonality, and adaptation to specific isolation source. Therefore, in this study, we selected *S*. Montevideo A as a model to conduct in-depth comparative genomic analysis aimed at identifying clades of differential public health concern and elucidating the mechanisms underlying the interplay between their enhanced or reduced human virulence, source -specific adaptation, and evolutionary trajectories.

**Figure 2:**
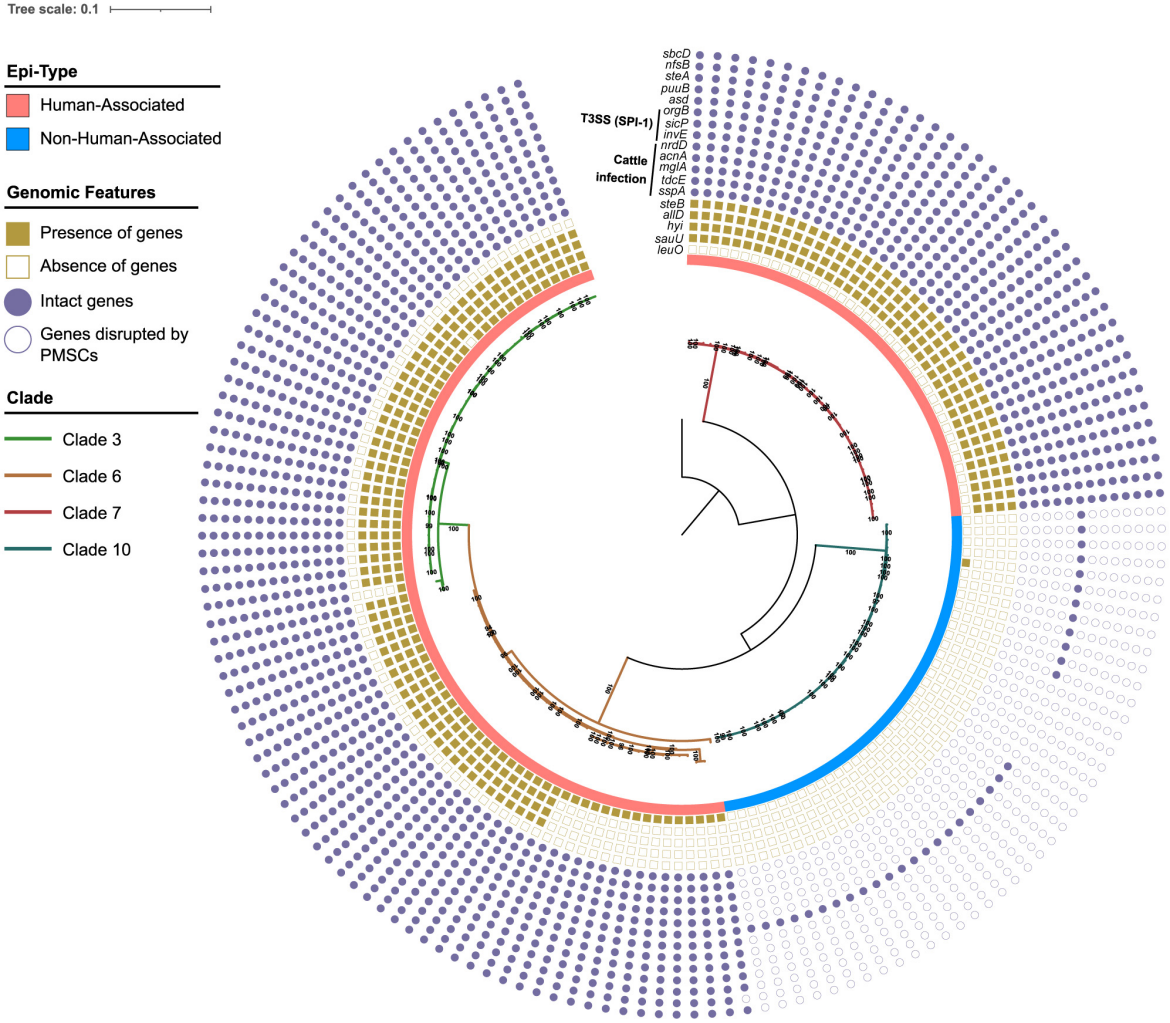
Phylogenetic relationship among human-associated and non-human-associated clades within *S*. Montevideo A. The maximum likelihood phylogeny was reconstructed from 31,240 core SNPs identified among representative isolates selected from each human-associated and non-human-associated clade for conducting comparative genomic analyses. A reference isolate of *S*. Oranienburg (assembly accession no. GCA_002062305) was included as an outgroup to root the tree, as this serovar has been reported to be phylogenetically closely related to *S*. Montevideo A. Branch lengths represent the average pairwise number of nucleotide substitutions per site. Clustering confidence (0–100) was assessed using 1,000 bootstrap repetitions, with values ≥70 displayed on corresponding branches. A comprehensive tree encompassing all 12 clades within Montevideo A has been presented in Chen et al. (2024).

Among the 12 clades within *S*. Montevideo A, clades 3, 6, and 7 showed a significant overrepresentation of human isolates (OR > 2; *p* < 0.05) and were classified as human-associated (“HA”) clades. Clade 10 showed a significant underrepresentation of human isolates (OR <0.5; *p* < 0.05) and was classified as a non-human-associated (“NHA”) clade (Table 1). The phylogenetic tree reconstructed using representative isolates from all HA and NHA clades revealed that clades 3 and 6 share a common ancestor (bootstrap support: 100), indicating a closer phylogenetic relationship between these two clades than with the other clades (Figure 2); this close relationship between clades 3 and 6 had been observed previously in a phylogenetic tree that included isolates representing all clades within *S*. Montevideo lineage A (Chen et al., 2024). Together with our previous findings that clades 10 and 7 were adapted to cattle and non-animal natural environments, respectively, whereas clades 3 and 6 lacked clear source-specific adaptation (Chen et al., 2024), our results here suggest that *S*. Montevideo A clades have undergone evolutionary divergence, leading to differential source-specific adaptation.

**Table 1:** Epi-type classification of clades within *S*. Montevideo A.

Clade	The United States			The United Kingdom			Final epi-type^d^
	# Isolates^a^	OR^b^	Epi-type^c^	# Isolates^a^	OR^b^	Epi-type^c^	
1	703	0.53	Neutral	115	0.68	Neutral	Neutral
2	0	NA^e^	NA	1	NA	NA	NA
3	92	6.13	HA	18	0.57	Neutral	HA
4	1	NA	NA	2	NA	NA	NA
5	2	NA	NA	3	NA	NA	NA
6	1,452	2.19	HA	144	1.22	Neutral	HA
7	1,360	2.69	HA	14	0.18	NHA	HA
8	52	21.80	HA	14	0.07	NHA	Neutral
9	0	NA	NA	8	NA	NA	NA
10	3,054	0.17	NHA	53	0.88	Neutral	NHA
11	44	1.14	Neutral	3	NA	NA	Neutral
12	0	NA	NA	1	NA	NA	NA

^a^Number of isolates in the NCBI Pathogen Detection database (Accessed August 31, 2021).

^b^Odds ratio for human isolates, defined as the odds of human isolates within a given clade relative to the odds of human isolates among all *Salmonella* isolates outside the clade.

^c^Epidemiological classification indicating association with human salmonellosis cases: HA, human-associated (OR > 2 and p-value <0.05); NHA, non-human-associated (OR <0.5 and p-value <0.05); Neutral, 0.5 ≤ OR ≤ 2 and/or p-value ≥ 0.05.

^d^Final epidemiological classification assigned following the decision tree depicted in Figure 1.

^e^Not applicable; the epidemiological classification was not determined due to insufficient ( ≤ 10) isolates collected from the corresponding country.

### Source-specific adaptation does not constrain pan-genome expansion among HA and NHA clades within *S*. Montevideo A

**Figure 3:**
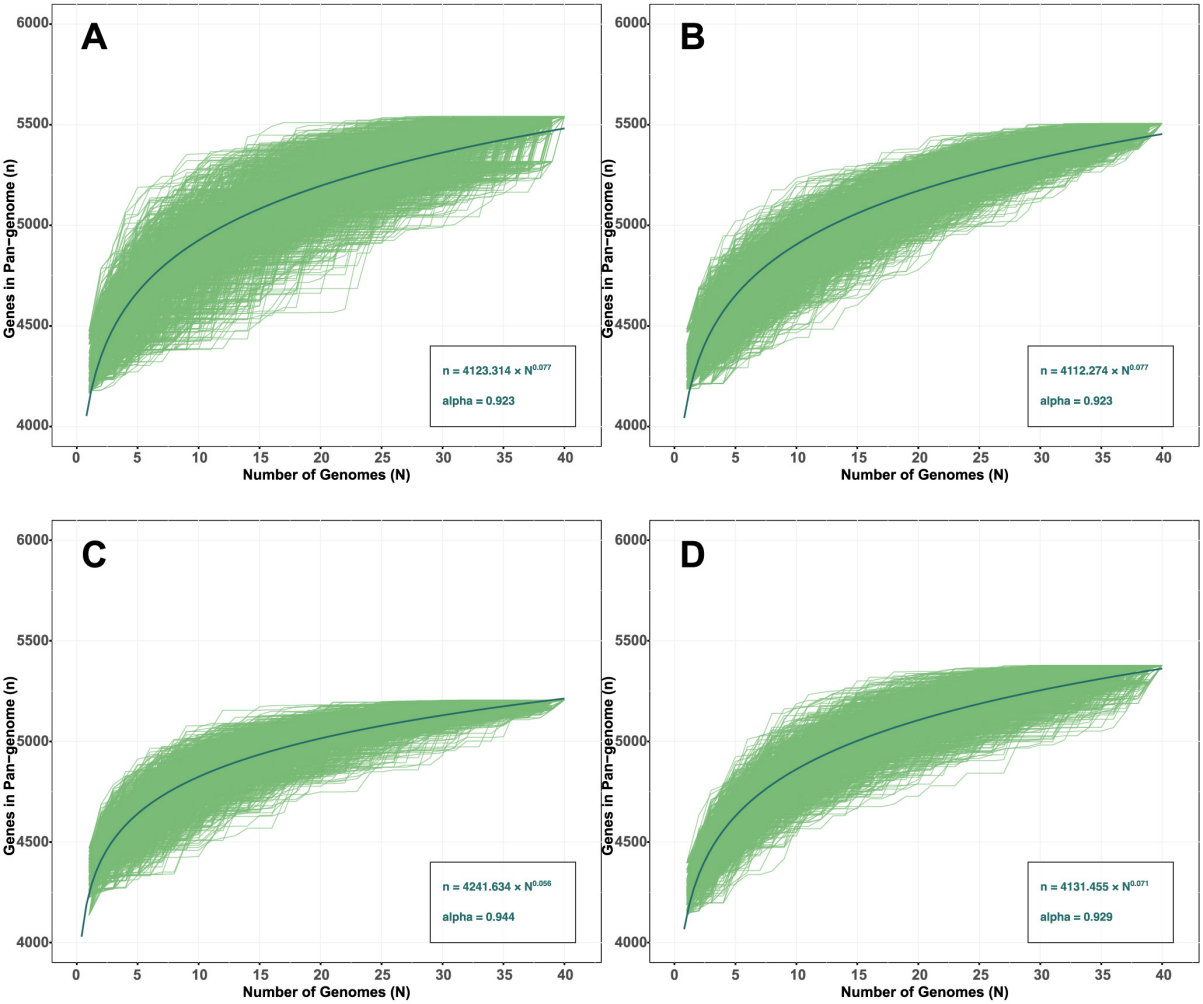
Rarefaction (pan-genome accumulation) curves and pan-genome openness for clades 3 **(A)**, 6 **(B)**, 7 **(C)**, and 10 **(D)**. The green curves represent 1,000 random permutations of genome addition order, while the blue curve denotes the mean rarefaction curve across permutations, showing the expected cumulative number of genes in the pan-genome as a function of the number of genomes analyzed.

To investigate whether the observed adaptation of clade 7 to non-animal natural environments and of clade 10 to cattle-related sources could be explained by a reduced pan-genome size and openness, we estimated the pan-genome size and assessed its openness for all four HA and NHA clades. Our results revealed variation in pan-genome size across clades, ranging from 5,206 genes in clade 7 to 5,542 in clade 3. Consistent with this, clades 7 and 3 showed the highest and lowest alpha values (0.944 and 0.923, respectively) among the four clades (Figure 3). Importantly, as the alpha values were <1 across all clades, our results indicated that each clade possessed an open pan-genome, suggesting that source-specific adaptation does not impose sufficient constraints to restrict pan-genome expansion. Therefore, isolates within each clade likely continue to engage in genetic exchange within their respective environment.

### Comparative genomics reveals clade-specific gene content variation and diverse contributions of mobile genetic elements (MGEs)

To enhance the mechanistic understanding of the differential human association in *S*. Montevideo A clades, we performed comparative genomic analysis between each HA clade and the NHA clade to identify genomic features potentially associated with enhanced or reduced human virulence. Compared to the NHA clade 10, a total of 174, 198, and 283 genes were significantly overrepresented in the HA clades 3, 6, and 7 (referred to as “HA genes”), respectively (Supplementary Table S3). Conversely, 160, 123, and 209 genes were significantly overrepresented in clade 10 when contrasted with clades 3, 6, and 7 (referred to as “NHA genes”), respectively. Particularly, we identified five chromosomal HA genes that could be mapped to known virulence factors (Table 2). These included three genes, *STM0520, hyi* (*STM0518*), and *allD* (*STM0528*), consistently overrepresented across all three HA clades; one gene, *STM0030*, overrepresented in clades 3 and 6; and one gene, *steB* (*STM1629*), encoding a type III secretion system effector, uniquely overrepresented in clade 7.

**Table 2:** Potential virulence factors significantly overrepresented in the human-associated (HA) clades 3, 6, and 7 relative to the non-human-associated (NHA) clade 10.

Gene	Gene product	Mapped virulence factors	Number of isolates (out of 40 total isolates) from each clade carrying the gene^a^			
			3	6	7	10
*STM0030*	LysR family transcriptional regulator LeuO	*PATRIC_VF^*b*^:* SL1344_0031	39	40	NS	1
*STM0520*	Putative sulfoacetate transporter SauU	*Victors^*c*^:* 16763900; *PATRIC_VF:* SL1344_0513	39	24	40	0
*hyi*	Hydroxypyruvate isomerase	*Victors:* 16763898; *PATRIC_VF:* SL1344_0511	39	24	40	0
*allD*	Ureidoglycolate dehydrogenase [NAD(+)]	*Victors:* 16763908; *PATRIC_VF:* SL1344_0521	39	24	40	0
*steB*	Type III secretion system effector SteB	*VFDB^*d*^*: VFG042214	NS	NS	40	0

^a^Presence of the gene is significantly associated with the indicated human-associated clades 3, 6 and 7 relative to the non-human-associated clade 10 based on Fisher's exact test with Benjamini–Hochberg correction (p <0.05). NS, not significant.

^b^Virulence factors annotated in the PATRIC (Pathosystems Resource Integration Center) database based on homology to known virulence genes.

^c^Victors database, a curated repository of experimentally verified bacterial virulence factors.

^d^Virulence Factor Database, a curated repository of experimentally verified bacterial virulence factors.

**Figure 4:**
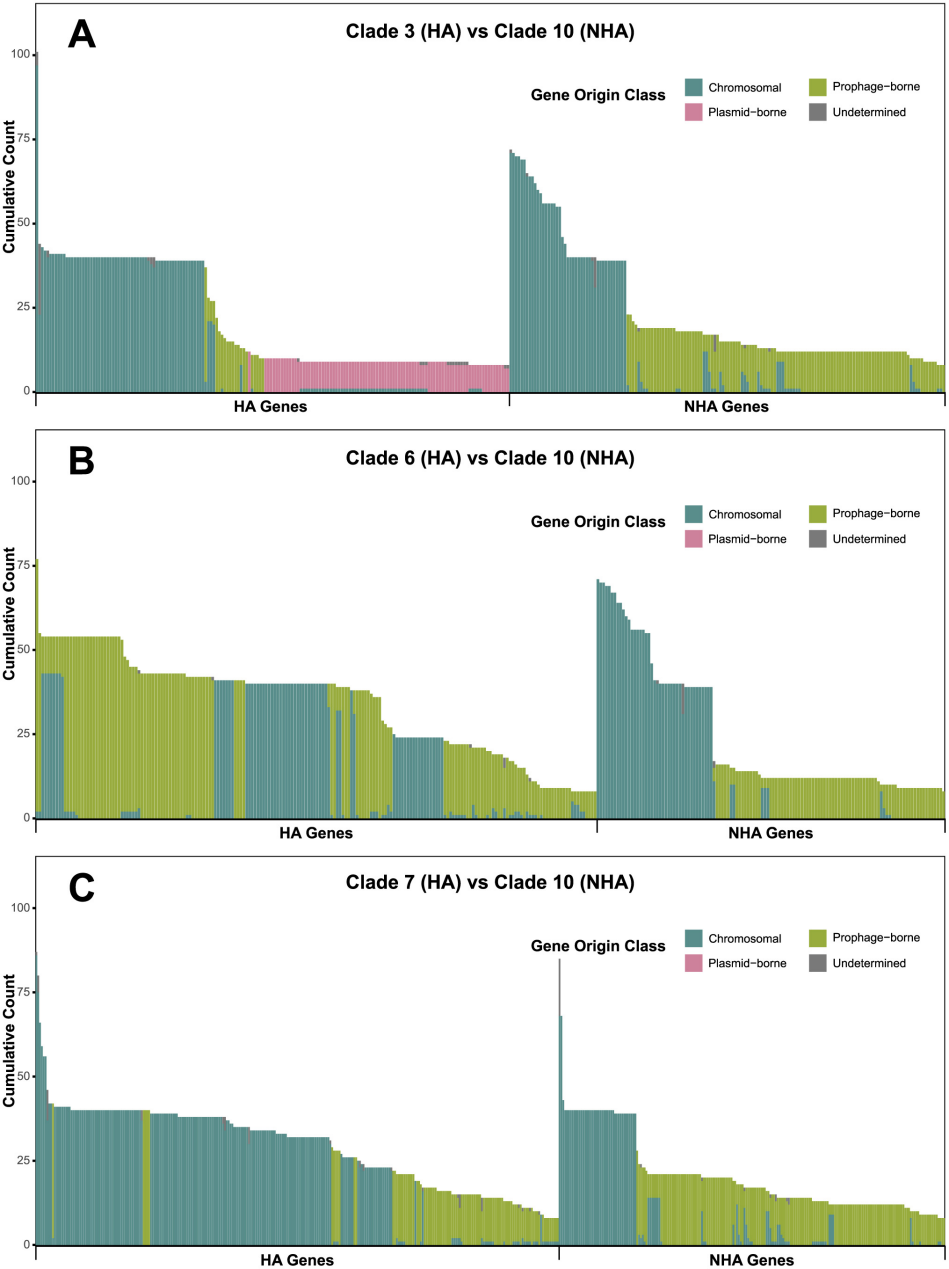
Genomic origin of genes significantly overrepresented in the human-associated clades (HA genes) or the non-human-associated clade (NHA genes), based on pairwise comparisons of clade 10 with clades 3 **(A)**, 6 **(B)**, and 7 **(C)**. Each stacked bar represents an individual gene, with its origin class displayed across all isolates in which the gene is present, providing the highest possible resolution. Genes were classified as “Chromosomal” if not associated with plasmids or prophages, “Plasmid-borne” if associated with plasmids but not prophages, “Prophage-borne” if associated with prophages but not plasmids, and “Undetermined” if they could not be unambiguously assigned to any of the preceding categories. Gene copies labeled as “refound” by Panaroo were also classified as “Undetermined.”

In addition to chromosomal genes, MGEs may be critical to acquisition of human virulence genes due to their capacity to rapidly disseminate adaptive traits across bacterial populations. Therefore, we sought to identify HA and NHA genes that likely originated from plasmids or prophages, the two major MGE classes. We found that genes located on plasmids or prophages accounted for 62.1%, 65.7%, and 35% of the HA genes in clades 3, 6, and 7, respectively, and for 69.4%, 61%, and 72.2% of the NHA genes in clade 10 when compared with clades 3, 6, and 7, respectively (Supplementary Table S3). Notably, clade 3 showed a higher proportion of plasmid-borne (52.3%) compared to prophage-borne (9.8%) HA genes (Figure 4). Further analysis revealed that these plasmid-borne genes correspond to an IncI1 plasmid carrying the IncI1.1 replicon, a plasmid type previously reported to contribute to virulence-related phenotypes in *Salmonella* (Kaldhone et al., 2019). In contrast, none of the HA genes in clades 6 and 7 originated from plasmids, and no NHA genes of plasmid origin were identified across any of the comparisons. These overall trends highlight the pivotal role of MGEs in driving the genomic diversification among the HA and NHA clades. Notably, although high proportions of HA genes were carried on MGEs, none could be mapped to known virulence factors, providing limited evidence supporting the contribution of MGEs to the presumed enhanced human virulence in the three HA clades.

### NHA-clade-specific accumulation of premature stop codons indicates functional genome disruption linked to cattle adaptation and attenuated human virulence

In contrast to the accessory genome, where genetic variation is characterized by the presence and absence of genes, variation within the core genome is typically represented by mutations that result in single nucleotide polymorphisms (SNPs). Notably, nonsense mutations, manifested as premature stop codons (PMSCs), often lead to truncated, dysfunctional protein products with potential phenotypic consequences, such as reduced virulence. Our results revealed a 7–10-fold higher number of PMSCs significantly overrepresented among the NHA and cattle-associated clade 10 compared to each of the HA clades (204 PMSCs in clade 10 vs. 29 in clade 3; 192 vs. 22 in clade 6; 170 vs. 17 in clade 7; Supplementary Table S4). In line with the overall trend, clade 10 showed a significant overrepresentation of 7–19-fold higher number of PMSCs that likely disrupt virulence factors compared to the HA clades (23 PMSCs in clade 10 vs. 3 in clade 3; 22 vs. 3 in clade 6; 19 vs. 1 in clade 7). Notably, we identified 13 disrupted genes that were significantly overrepresented in clade 10 compared to all HA clades (Table 3, Figure 2). Five of these genes were associated with calf infections, including one gene (*sspA*) required for lethal infections and four genes (*tdcE, mglA, acnA*, and *nrdD*) implicated in intestinal colonization. The presence of PMSCs in genes involved in calf infection supports the hypothesis that clade 10 may have adapted to become a commensal strain with cattle. In addition, three disrupted genes (*invE, sicP, orgB*) were linked to the Type III Secretion System (T3SS) encoded by *Salmonella* Pathogenicity Island 1 (SPI-1). Collectively, these findings provide mechanistic insights at the molecular level into the adaptation of clade 10 to cattle, possibly accompanied by a reduction in human virulence.

**Table 3:** Premature stop codons (PMSCs) in virulence factors that were significantly overrepresented in the non-human-associated (NHA) clade 10.

Position in the reference genome^a^	Disrupted gene	Disrupted gene coordinates	Truncated gene proportion^b^	Gene product	Mapped virulence factors	Overrepresented when compared to clade			Remarks
						3	6	7	
357,199	*asd*	Forward: 356357–357463	0.76	Aspartate-semialdehyde dehydrogenase	*Victors:* 267995764 *PATRIC_VF:* STM14_4260	Yes	Yes	Yes	
547,668	*sspA*	Forward: 547213–547851	0.71	Stringent starvation protein A	*Victors:* 16766637 *PATRIC_VF:* STM14_4033	Yes	Yes	Yes	Associated with lethal infection in cattle
648,108	*tdcE*	Forward: 647740–650034	0.16	Formate C-acetyltransferase	*Victors:* 15803653 *PATRIC_VF:* Z4466	Yes	Yes	Yes	Associated with intestinal colonization in cattle
649,170	*tdcE*	Forward: 647740–650034	0.62	Formate C-acetyltransferase	*Victors:* 15803653 *PATRIC_VF:* Z4466	Yes	Yes	Yes	Associated with intestinal colonization in cattle
775,723	*puuB*	Forward: 775262–776563	0.35	FAD-binding oxidoreductase	*Victors:* 16766428 *PATRIC_VF:* SL1344_3102	Yes	Yes	Yes	
1,018,700	*invE*	Forward: 1018635–1019753	0.06	Type III secretion system outermembrane contact sensing protein (gatekeeper) InvE; invasion protein	*Victors:* 16766203 *VFDB*: VFG000558 *PATRIC_VF:* SL1344_2876, STM2897	Yes	Yes	Yes	Associated with SPI-1-encoded T3SS
1,036,504	*sicP*	Forward: 1036145–1036537	0.92	Chaperone protein SicP (chaparone for SptP)	*VFDB*: VFG000541 *PATRIC_VF:* SL1344_2859	Yes	Yes	Yes	Associated with SPI-1-encoded T3SS
1,046,130	*orgB*	Forward: 1045756–1046427	0.56	Oxygen-regulated invasion protein OrgB; associated with InvC ATPase of type III secretion	*Victors:* 16766175 *VFDB*: VFG000532 *PATRIC_VF:* STM14_3468, SL1344_2849	Yes	Yes	Yes	Associated with SPI-1-encoded T3SS
1,047,387	*hilC*	Forward: 1047241–1048128	0.16	Type III secretion transcriptional regulator HilC (= SirC)	*Victors:* 16766173 *PATRIC_VF:* SL1344_2847	No	No	Yes	Associated with SPI-1-encoded T3SS
1,331,016	*sinH*	Forward: 1329595–1331787	0.65	Intimin-like inverse autotransporter SinH	*VFDB*: VFG002307	Yes	Yes	No	
1,678,319	*mglA*	Forward: 1677027–1678547	0.85	Galactose/methyl galactoside ABC transporter ATP-binding protein MglA	*Victors:* 15802705 *PATRIC_VF:* Z3404	Yes	Yes	Yes	Associated with intestinal colonization in cattle
2,037,555	*fadD*	Forward: 2036929–2038680	0.36	Long-chain-fatty-acid–CoA ligase FadD	*Victors:* 16765159 *PATRIC_VF:* SL1344_1747	Yes	Yes	No	
2,038,185	*fadD*	Forward: 2036929–2038680	0.72	Long-chain-fatty-acid–CoA ligase FadD	*Victors:* 16765159 *PATRIC_VF:* SL1344_1747	Yes	Yes	No	
2,146,696	*acnA*	Forward: 2146004–2148679	0.26	aconitate hydratase AcnA	*Victors:* 15801901	No	No	Yes	Associated with intestinal colonization in cattle
2,147,728	*acnA*	Forward: 2146004–2148679	0.64	aconitate hydratase AcnA	*Victors:* 15801901	Yes	Yes	Yes	Associated with intestinal colonization in cattle
2,290,492	*steA*	Forward: 2290070–2290702	0.67	Type III secretion system effector protein SteA	*Victors:* 16764927 *VFDB*: VFG042067 *PATRIC_VF:* SL1344_1514	Yes	Yes	Yes	
2,351,034	*yneE*	Reverse: 2351828–2350881	0.84	UPF0187 protein YneE	*Victors:* 16764872 *PATRIC_VF:* STM474_1538	Yes	Yes	No	
2,459,454	*ssaT*	Reverse: 2459636–2458857	0.23	SPI-2 type III secretion system minor export apparatus protein SsaT	*VFDB*: VFG000521 *PATRIC_VF:* STM474_1427	Yes	Yes	No	Associated with SPI-2-encoded T3SS
2,718,621	*solA*	Forward: 2718355–2719473	0.24	N-methyl-L-tryptophan oxidase SolA	*Victors:* 16764516 *PATRIC_VF:* SL1344_1097	No	No	Yes	
2,852,048	*aroA*	Reverse: 2853226–2851943	0.92	3-phosphoshikimate 1-carboxyvinyltransferase	*PATRIC_VF:* STM0978, SL1344_0915, STM14_1106, SEN0882 *Victors:* 82777572	No	No	Yes	
3,234,147	*fepA*	Forward: 3232970–3235225	0.52	TonB-dependent siderophore receptor; outer membrane receptor for ferric enterobactin and colicins B, D	*PATRIC_VF:* STM14_0682, STM474_0605 *Victors:* 16763962	Yes	Yes	No	
3,239,142	*nfsB*	Forward: 3239104–3239757	0.06	Oxygen-insensitive NAD(P)H nitroreductase	*Victors:* 16763955 *PATRIC_VF:* SL1344_0566	Yes	Yes	Yes	
3,357,695	*cyoB*	Forward: 3357456–3359447	0.12	Cytochrome O ubiquinol oxidase subunit I	*Victors:* 16763823 *PATRIC_VF:* SL1344_0436	No	No	Yes	
3,400,370	*sbcD*	Forward: 3399336–3400538	0.86	Exonuclease subunit SbcD	*Victors:* 16763776 *PATRIC_VF:* STM474_0413	Yes	Yes	Yes	
3,466,981	*stbC*	Forward: 3466319–3468880	0.26	Fimbrial outer membrane usher protein	*PATRIC_VF:* STM474_0353 *Victors:* 16763718	Yes	Yes	No	
3,468,616	*stbC*	Forward: 3466319–3468880	0.90	Fimbrial outer membrane usher protein	*PATRIC_VF:* STM474_0353 *Victors:* 16763718	No	No	Yes	
3,770,491	*carB*	Reverse: 3773082–3769855	0.80	Carbamoyl-phosphate synthase large subunit	*PATRIC_VF:* Z0038 *Victors:* 16128027	Yes	Yes	No	
3,846,023	*arcA*	Forward: 3845769–3846485	0.35	Two-component system response regulator ArcA	*Victors:* 218927655	Yes	Yes	No	
4,012,263	*nrdD*	Forward: 4011685–4013823	0.27	Anaerobic ribonucleoside-triphosphate reductase	*Victors:* 15804828	Yes	Yes	Yes	Associated with intestinal colonization in cattle

^a^The reference genome is available in GenBank under accession number GCA_005576575.1.

^b^The truncated gene proportion was calculated as the ratio of the sequence length from the gene start to the PMSC to the full gene length.

### Divergent evolutionary trajectories of HA and NHA clades reflect the accelerated evolution and cattle adaptation of clade 10

**Figure 5:**
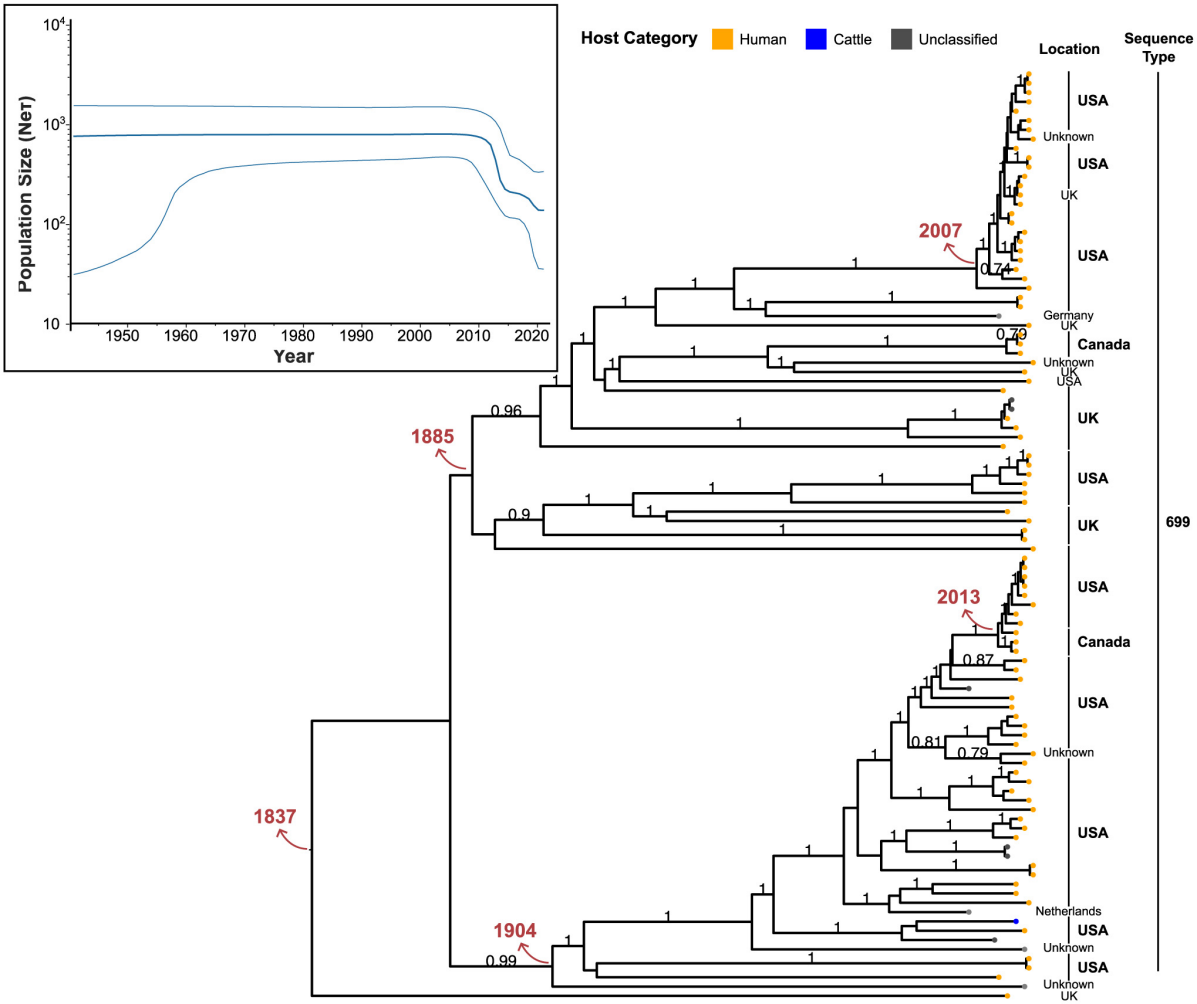
Time-scaled phylogeny and Bayesian Skyline plot (BSP) for clade 3. The Bayesian time-scaled maximum clade credibility (MCC) phylogeny and BSP were reconstructed using core SNPs identified among 100 representative isolates. The inset displays the BSP, depicting changes in effective population size over time, where the *y*-axis represents the product of effective population size (*Ne*) and generation time (τ), and the *x*-axis represents time in years. The bold line indicates the median of *Ne*τ following the timeline, while the area between the two surrounding lines denotes the 95% highest posterior density (HPD) interval. The MCC phylogeny adjacent to the BSP is time-scaled from left to right, with branch lengths reported in years. The most recent common ancestors of the entire clade, two major subclades, and two emerging clusters are labeled with their respective emergence years in red. Posterior probabilities of branch support are shown on the corresponding branches. Where available, the following information are displayed next to the leaf nodes: (i) seven-gene multi-locus sequence type and (ii) geographical location.

**Figure 6:**
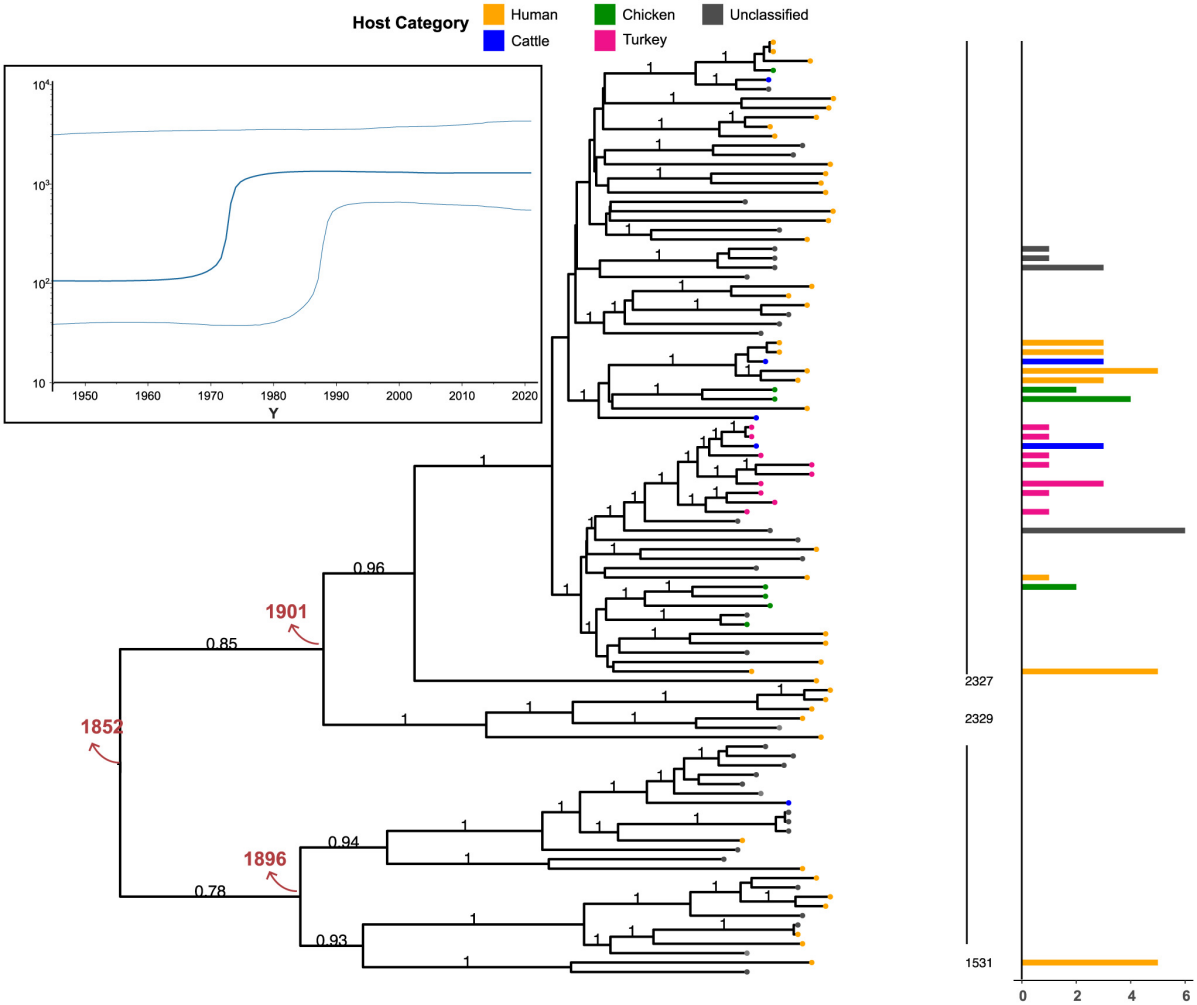
Time-scaled phylogeny and Bayesian Skyline plot (BSP) for clade 6. The Bayesian time-scaled maximum clade credibility (MCC) phylogeny and BSP were reconstructed using core SNPs identified among 100 representative isolates. The inset displays the BSP, depicting changes in effective population size over time, where the *y*-axis represents the product of effective population size (*Ne*) and generation time (τ), and the *x*-axis represents time in years. The bold line indicates the median of *Ne*τ following the timeline, while the area between the two surrounding lines denotes the 95% highest posterior density (HPD) interval. The MCC phylogeny adjacent to the BSP is time-scaled from left to right, with branch lengths reported in years. The most recent common ancestors of the entire clade and two major subclades are labeled with their respective emergence years in red. Posterior probabilities of branch support are shown on the corresponding branches. Where available, the following information are displayed next to the leaf nodes: (i) seven-gene multi-locus sequence type and (ii) geographical location. The bar plot on the far-right displays, for each representative isolate, the number of antimicrobial resistance (AMR) classes associated with it, with bars color-matched to the corresponding leaf nodes.

The evolutionary analysis of individual HA and NHA clades revealed notable variation in both emergence time and evolutionary rate across clades (Table 4). Clade 10, which emerged most recently (circa 1905), showed the highest evolutionary rate (1.9 × 10^−7^ substitutions/site/year) among all clades analyzed, potentially due to its commensal association with cattle, which may facilitate a shorter generation time within the animal reservoir. This accelerated rate likely contributed to the extensive accumulation of PMSCs in this clade, including those affecting key virulence factors. Importantly, 59% of the representative isolates from clade 10 originated from cattle-related sources, spanning 17 states in the US and Mexico and encompassing diverse sample types including animals, feces, tissue/organs, food products, feed, and farm or processing environments (Supplementary Table S5). These findings suggest a rapid clonal expansion of clade 10, possibly facilitated by its adaptation to cattle-related sources and the concomitant reduction in virulence. In contrast, the non-animal natural environment-adapted clade 7 emerged circa 1744 Before Common Era (BCE) and exhibited a markedly lower evolutionary rate (8.1 × 10^−9^) than the other clades, suggesting long-term genomic stability under minimal exposure to animal hosts, likely reflecting a longer generation time in a more variable natural environment. The two HA clades lacking source associations were both estimated to have emerged within one century prior to clade 10 (i.e., circa 1837 for clade 3 and circa 1852 for clade 6) and showed intermediate evolutionary rates (8.9 × 10^−8^ for clade 3 and 1.1 × 10^−7^ for clade 6). Clade 3 contained two major subclades, emerging circa 1885 and 1904, respectively, both of which predominantly comprised pan-susceptible human isolates from the US (Figure 5). Notably, each subclade gave rise to a recent common ancestor (emerging circa 2007 and 2013, respectively), followed by an increase in short branches and coalescent events—hallmarks of reduced genetic diversity (i.e., population bottleneck)—that closely corresponded with the decline in effective population size around 2010 in the Bayesian Skyline plot (BSP; Figure 5). Importantly, these two emerging clusters consisted of isolates from the US and Canada that were not linked to any reported outbreaks, suggesting ongoing clonal expansions. Such expansions may ultimately increase the genetic diversity and overall effective population size of clade 3, posing elevated public health concerns associated with this HA clade. Clade 6 comprised two major subclades that exhibited genetic and geographic segregation. One subclade (emerging circa 1896) predominantly consisted of pan-susceptible ST195 isolates from Europe and the Middle East, whereas the other subclade (emerging circa 1901) predominantly comprised ST4 isolates from the US, with 29.3% of its isolates having acquired antibiotic resistance (Figure 6). Unlike clade 3, no evidence of recent clonal expansion was observed in clade 6. Notably, our findings revealed the emergence of five clades or major subclades between 1885 and 1905, suggesting the presence of systematic triggers that may have driven their emergence and subsequent evolution.

**Table 4:** Key evolutionary parameters of the human-associated (HA) and non-human-associated (NHA) clades.

Clade	Epi-type classification^a^	Source association	Mean TMRCA in years (95% HPD interval)^b^	Mean evolutionary rate in substitutions/site/year (95% HPD interval)^c^	Estimated emergence year^d^
3	HA	None	184.3 (81–323.8)	8.9 × 10^−8^ (4.9 × 10^−8^-1.3 × 10^−7^)	1837
6	HA	None	169.4 (76.4–299.1)	1.1 × 10^−7^ (4.6 × 10^−8^-1.7 × 10^−7^)	1852
7	HA	Non-animal environment	3,764.6 (947–8,090.9)	8.1 × 10^−9^ (1.8 × 10^−9^-1.6 × 10^−8^)	1744 BCE
10	NHA	Cattle	115.8 (63.5–180.4)	1.9 × 10^−7^ (1.3 × 10^−7^-2.5 × 10^−7^)	1905

^a^HA: human-associated; NHA: non-human-associated.

^b^The mean and 95% highest posterior density (HPD) of time to a most recent common ancestor (TMRCA) were inferred from the parameter “TreeHeight,” estimated by Tracer.

^c^The mean and 95% HPD of evolutionary rate were inferred from the parameter “rate.mean,” estimated by Tracer.

^d^The estimated emergence year of a given clade was calculated by subtracting the mean TMRCA from 2021, the collection year of the most recent isolate in the analysis.

## Discussion

*S*. Montevideo is a diverse serovar commonly associated with bovines in the US (Blau et al., 2005; USDA-APHIS, 2005; Bosilevac et al., 2009; Valenzuela et al., 2017; Craig et al., 2024). However, few beef and dairy linked outbreaks have been associated with this serovar (Jackson et al., 2013; Gould et al., 2014; Laufer et al., 2015; Canning et al., 2023; Marshall et al., 2024). In this study, we identified clades within *S*. Montevideo A that show a significant over- or underrepresentation of human isolates, and carried out comparative genomics and evolutionary analyses to elucidate the interplay among presumptive hypo- or hypervirulence, niche adaptation, and evolution of the target clades. We used genome data and metadata from isolates available on NCBI PD. We acknowledge that NCBI PD is a non-random surveillance resource, with overrepresentation of US and UK isolates and potential biases arising from outbreak-driven sampling. To partially mitigate these effects in epi-type classification, we restricted analyses to US and UK isolates and applied stringent OR thresholds (>2 and <0.5), in addition to statistical significance, to define associations more robust to sampling biases. Future studies should focus on more systematic approaches to address these limitations. Our results indicate that human isolates are significantly overrepresented among three clades (3, 6, and 7) within the predominant phylogenetic lineage of *S*. Montevideo, whereas human isolates are significantly underrepresented in the cattle-associated clade 10. Clades 3 and 6 emerged at similar time, circa 200 years ago. However, while clade 6 has shown no distinguishable populational characteristics since its emergence, clade 3 shows evidence of a recent reduction in genetic diversity, reflecting either a population bottleneck or the early stage of clonal expansion of a potentially better-adapted subtype. Clade 10 genomes harbor multiple PMSCs in virulence genes, which may contribute to their reduced association with human infections. We also identified clade-specific differences driven by mobile genetic elements, including plasmids and prophages, that distinguish clades 3, 6, and 7 from one another and from clade 10. Finally, virulence genes present in clades 3, 6 and 7, but completely absent in clade 10, may underline the possible enhanced virulence potential of these clades. In the following sections, we further examine the relative contributions of mobile and chromosomal genetic elements to clade-specific genomic differentiation and virulence, the adaptive signatures underlying cattle specialization and potential virulence attenuation in clade 10, and the broader evolutionary trajectories and public health implications of these divergent clades.

### While MGEs represent potential modulators for clade-specific genomic diversification within *S*. Montevideo A, genes implicated in virulence were chromosomally-located

Most genes found to be overrepresented in HA clades 3 and 6 as compared to the NHA clade 10 could be mapped to MGEs (i.e., plasmids and prophages). The role of MGE in the diversification of strains has been well-documented (Vassallo et al., 2020; Weisberg and Chang, 2023; Weisberg et al., 2023; Tokuda and Shintani, 2024), as each MGE can carry tens to hundreds of genes. Interestingly, plasmids and prophages were responsible for the overrepresentation of most genes associated with clades 3 and 6, respectively, evidencing that these two HA clades evolved independently to reach similar epidemiological characteristics. Conversely, most genes overrepresented among the HA clade 7 isolates were located on the chromosome, followed by genes located in prophages. Hence, plasmid-borne genes were rarely overrepresented among clade 7 isolates. Although MGE accounted for most HA-associated genes, the five potential virulence genes overrepresented among HA clades were located in the chromosome of the isolates. Three of these five genes (STM0520, *hyi*, and *allD*) were overrepresented in all HA clades. These three genes encode (i) a putative permease belonging to the major facilitator superfamily of transporter proteins (STM0520), (ii) a hydroxypyruvate isomerase (*hyi;* also known as *gip*, STM0518), and (iii) an ureidoglycolate dehydrogenase involved in the utilization of allantoin as a nitrogen source (Matiasovicova et al., 2007) (*allD;* STM0528). All three of these genes have been reported to contribute to long-term systemic infection in mice using a *S*. Typhimurium model (Lawley et al., 2006). In addition, *hyi* has been reported as being preferentially expressed by *S*. Enteritidis in the primary chicken oviduct epithelial cells for reproductive-tract colonization of chicken (McKelvey Jessica et al., 2014). The mechanisms by which these three genes influence *Salmonella* virulence remain, however, unknown. One gene, STM0030, was overrepresented in HA clades 3 and 6, but not 7. STM0030 encodes a LysR-family transcriptional regulator that has been previously reported to contribute to *S*. Typhimurium growth inside macrophages and to virulence in mice (Zhang et al., 2019). Moreover, deletion of STM0030 in *S*. Typhimurium resulted in downregulation of allantoin utilization genes (Zhang et al., 2019), possibly linking the function of STM0030 to that of *allD*. Conversely, another gene, *steB* (STM1629), was overrepresented only in the HA clade 7, but not in clades 3 and 6. *steB* encodes a type III secretion system effector protein SteB (Geddes et al., 2005), which has not been fully characterized (Worley, 2025), but has been implicated in intestinal disease, as it is found in *Salmonella* serovars associated with gastroenteritis, but is usually absent in those causing extraintestinal infections, such as *S*. Typhi and *S*. Paratyphi A (Jennings et al., 2017). Therefore, the overrepresentation of some potential virulence genes among isolates from HA clades 3, 6, and 7, supports that isolates from these HA clades may be more likely to cause human illness than isolates from the NHA clade. Further investigation, however, will be needed to elucidate the mechanistic roles these virulence genes play in enhancing the virulence of *S*. Montevideo isolates within the HA clades.

### Clade 10 appears to have evolved into a virulence-attenuated cattle commensal clade

Host adaptation in *Salmonella* is often facilitated through specific gene acquisitions coupled with functional gene losses, the latter typically manifested as an accumulation of disrupted coding sequences (Foley Steven et al., 2013; Langridge et al., 2015; Sandholt et al., 2021; Cohn et al., 2022). Host-restricted or host-adapted *Salmonella* serotypes have been shown to harbor a higher number of nonfunctional genes compared to generalist serotypes (Wang et al., 2024), and a similar trend has been observed when comparing host-adapted lineages with generalist lineages within the same serotype (Cohn et al., 2022). In particular, Cohn et al. (2022) identified a large clonal ST367 clade within the predominant *Salmonella* Cerro lineage that appears cattle-adapted, and that cattle-derived isolates harbored significantly more disrupted coding sequences than those from other sources. Consistent with this, we found that, compared to individual HA clades, the cattle-adapted NHA clade 10 showed a significant overrepresentation of PMSCs in the core genome and, more specifically, in virulence genes. Notably, *sspA*, encoding the stringent starvation protein A (SspA), was disrupted in all isolates from the NHA clade 10 but remained intact in all isolates across the three HA clades. SspA is an SPI-1 T3SS-secreted effector that contributes to host internalization of *S*. Typhimurium through its actin-binding activity and supports intracellular replication by modulating the morphology and positioning of *Salmonella*-containing vacuoles (SCVs) in concert with SPI-2 T3SS-secreted effectors (Brawn et al., 2007; Pillay et al., 2023). Experiments using calf infection models have demonstrated that SspA is essential for lethal systemic disease, as calves infected with a *S*. Typhimurium *sspA* mutant strain developed diarrhea but did not succumb to fatal outcomes (Tsolis et al., 2000). Furthermore, PMSCs in *tdcE, mglA, acnA*, and *nrdD* were identified exclusively in isolates from clade 10. Inactivation of these four genes has been shown to reduce the fitness of enterohemorrhagic *Escherichia coli* and *S*. Typhimurium during intestinal colonization in calves (van Diemen et al., 2005; Chaudhuri et al., 2013), suggesting further attenuation of host morbidity and inflammatory responses. Finally, compared to all HA clades, clade 10 showed a significant overrepresentation of PMSCs in genes encoding integral components of the SPI-1-encoded T3SS (*invE, sicP*, and *orgB*), which mediates epithelial cell invasion and early-stage virulence. InvE regulates protein translocation by controlling Sip (*Salmonella* invasion protein) translocases that deliver bacterial proteins into host epithelial cells to modulate cellular functions (Kubori and Galan, 2002); SicP acts as a dedicated chaperone stabilizing and promoting the translocation of SptP, an effector that facilitates *Salmonella* uptake by reversing cytoskeletal changes and supports replication within SCVs (Fu and Galán, 1998, 1999); OrgB is a key structural element of the SPI-1 T3SS sorting platform (Lou et al., 2019). Consequently, disruption of these three genes likely impaired the ability of clade 10 isolates to invade intestinal epithelial cells and deliver virulence factors, thus attenuating early-stage host interactions and infections. Taken together, the accumulation of PMSCs in diverse loci implicated in epithelial invasion and systemic infection suggests that the cattle-adapted clade 10 may be undergoing an evolutionary transition away from acute pathogenicity and toward a strategy favoring persistent, subclinical carriage in cattle. Such a shift may facilitate clade 10's continued adaptation to cattle-related sources by minimizing host morbidity and immune activation, while enhancing long-term persistence and transmission within cattle populations. In line with this, *S*. Montevideo has been reported as the most prevalent serovar in intestinal samples and peripheral lymph nodes of healthy cattle across different studies (Webb et al., 2017; Bonifait et al., 2021).

### Some *S*. Montevideo shows divergent evolutionary trajectories that may impact their relevance to public health

Our analyses suggest that four clades within *S*. Montevideo's predominant lineage have undergone divergent evolution, likely shaped by distinct selective pressures encountered in diverse ecological niches and leading to different source adaptations and ability to cause illness.

The non-animal natural environment-adapted clade 7 showed the lowest evolutionary rate among the four clades, suggesting long-term persistence in environmental reservoirs with limited exposure to animal hosts. In fact, this clade showed the lowest evolutionary rate when compared to 11 animal-associated clades from seven *Salmonella* serovars in our previous study (Chen et al., 2024). In natural environments such as water, soil, and plant surfaces, *Salmonella* is frequently subjected to unpredictably harsh conditions including nutrient scarcity, temperature fluctuations, UV exposure, and desiccation. A direct consequence of this may be an extended generation time and even entry into dormancy with infrequent replications, yielding a slower molecular clock (Finn et al., 2013; Lebre et al., 2017; DasSarma and DasSarma, 2018). Furthermore, *Salmonella* thriving in such environments may face strong purifying selection to maintain fitness under a broad range of environmental stresses, further reducing the evolutionary rate. Notably, certain mechanisms used by *Salmonella* to survive in non-animal natural environments may mirror those used during infection (Brown et al., 2021), and virulence factors have been implicated in environmental persistence. For instance, *sopD* and *sseD* have been demonstrated to play a pivotal role in desiccation tolerance and survival (Maserati et al., 2017). Consistent with this, clade 7 showed a significant overrepresentation of human isolates, coupled with few disrupted virulence factors in comparison to the cattle-adapted clade 10, thus possibly representing a latent environmental hazard capable of causing infections when transmitted to humans through contaminated produce or water. Given that produce accounts for most multistate outbreaks linked to *Salmonella* (Liu et al., 2018), our characterization of clade 7 highlights the need for environmentally inclusive surveillance, especially in light of climate change and possible water reuse practices that may increase human exposure to environmentally persistent pathogens.

Although the most recent among the four clades, the cattle-adapted NHA clade 10 showed the highest evolutionary rate, with a disproportionate accumulation of PMSCs, including some that may be linked to attenuated fitness during gut colonization, invasion, and intracellular survival. This accelerated evolutionary rate may reflect an interplay of relaxed purifying selection, neutral drift, and adaptive loss of functional genes during or following the clade's adaptation to cattle-related sources (Holt et al., 2009). Relaxed purifying selection has been observed in fish-adapted strains of *Streptococcus agalactiae* and in *Salmonella* serovars Paratyphi A and Typhi (Holt et al., 2009; Rosinski-Chupin et al., 2013), likely due to reduced selective pressures within stable host-associated environments (Nuccio and Baumler, 2014). In contemporary intensive cattle production systems, *Salmonella* transmission can be recurrent through fecal-oral routes, contaminated feed, water troughs, and shared housing, and may be further facilitated by crowding and inadequate hygiene, promoting extensive circulation within and across herds (Bentum et al., 2025). Such transmission dynamic, coupled with subclinical carriage in apparently healthy hosts, may lessen the need for environmental resilience or broad host tropism, relaxing purifying selection pressures and fostering neutral drift among isolates from clade 10. On the other hand, studies have also demonstrated that gene function loss may be linked to increased fitness and adaptation to specific hosts, indicating selective rather than neutral processes. For instance, Langridge et al. (2015) showed that many pseudogenes in host-adapted *Salmonella* serovars cluster in functional categories such as membrane surface structure, metabolism pathways, and transport systems. Similarly, Holt et al. (2009) found the independent accumulation of similar pseudogenes in *S*. Typhi and *S*. Paratyphi A, suggesting convergent evolution driven by adaptive gene loss. Both *S*. Montevideo HA clades 3 and 6 showed intermediate evolutionary rates, falling between the long-term genomic stability of clade 7 and the accelerated evolution of clade 10. Clade 6 comprises isolates from diverse animal and environmental sources and harboring a range of antimicrobial resistance determinants. Moreover, isolates from *S*. Montevideo clade 6 were linked to a multistate outbreak associated with whole cucumbers in the US, which lasted until May 2025 (Centers for Disease Control and Prevention, 2025), underscoring the substantial public health relevance of this clade. Despite the close phylogenetic relatedness to clade 6, clade 3 comprises almost exclusively pan-susceptible human isolates. Notably, clade 3 contains two independent clusters emerging ca. 2010, both showing hallmarks of recent clonal expansion without association to reported outbreaks. This indicates that clade 3 is undergoing concurrent diversification processes, potentially leading to an overall rise in genomic diversity and effective population size, possibly posing greater risks of generating emergent, epidemiologically fit variants. Importantly, clonal expansion reported in *Salmonella* lineages across diverse serovars has typically been attributed to host adaptation, acquisition of antimicrobial resistance, and/or clonal replacement (Kingsley and Baumler, 2000; Butaye et al., 2006; Kingsley et al., 2009; Bawn et al., 2020; Chen et al., 2024). The expansion of the two clusters within clade 3 thus represents a rare instance where clonal expansion is unlikely to be driven by these factors. Rather, the potential epidemiological success of clade 3 and its expanding clusters may reflect demographic founder effects, geographically dispersed or low-level endemic transmission, and enhanced transmission efficiency, factors that are more consistent with surveillance blind spots than limited epidemiological impact. At the time of this study, clade 3 comprises only 121 isolates in the NCBI PD database, the majority of which are assigned to sequence type ST699. This is substantially fewer than the number for the other clades (1,727 for clade 6, 1,442 for clade 7, and 3,211 for clade 10), indicating that clade 3 currently represents a rare subtype. Nevertheless, the observed expansion dynamics suggest a capacity for rapid population growth in the coming years. As clade 3 continues to increase in population size and diversify its genetic repertoire, the likelihood of adaptive shifts, such as enhanced virulence, persistence, or antimicrobial tolerance, may intensify.

Hence, our findings underscore the need for a risk-based approach to strategies to control *Salmonella* in the environment and food products as not all *Salmonella* seem to present the same likelihood of causing human illnesses. As previously described for *S*. Kentucky (Le Hello et al., 2011; Richards et al., 2023; Chen et al., 2024) and *S*. Cerro (Cohn et al., 2022; Chen et al., 2024), *S*. Montevideo shows intra-serotype diversity that may impact the public health significance of different strains within this serotype.

## Data Availability

The original contributions presented in the study are included in the article/Supplementary material, further inquiries can be directed to the corresponding author.
